# Impact of Periodonto-pathogenic Microbiota and Sociodemographic Variables on Periodontal Status during Pregnancy and Postpartum Period

**DOI:** 10.3290/j.ohpd.a45355

**Published:** 2020-10-02

**Authors:** Zhen Huang, Brett P. DerGarabedian, Lu He, Yueqin Sha, Zhibin Chen, Jun Kang, Yu Cai, Ping Gao

**Affiliations:** a Periodontist, Department of Periodontology, Peking University School and Hospital of Stomatology and National Clinical Research Center for Oral Diseases; National Engineering Laboratory for Digital and Material Technology of Stomatology; Beijing Key Laboratory of Digital Stomatology, Beijing, Beijing, China. Clinical data collection, conducted the experiments, analysed the results, prepared and reviewed the manuscript.; b PhD Student, Department of Periodontics, School of Dental Medicine, University of Pennsylvania, Philadelphia, PA, USA. Analysed the results, revised and reviewed the manuscript.; c Professor, Department of Periodontology, Peking University School and Hospital of Stomatology and National Clinical Research Center for Oral Diseases; National Engineering Laboratory for Digital and Material Technology of Stomatology; Beijing Key Laboratory of Digital Stomatology, Beijing, Beijing, China. Designed and interpreted the experiments, clinical data collection, revised and reviewd the manuscript.; d Professor, Department of Periodontology, Peking University School and Hospital of Stomatology and National Clinical Research Center for Oral Diseases; National Engineering Laboratory for Digital and Material Technology of Stomatology; Beijing Key Laboratory of Digital Stomatology, Beijing, Beijing, China. Designed the experiments, reviewed the manuscript.; e Periodontist, Department of Periodontology, Peking University School and Hospital of Stomatology and National Clinical Research Center for Oral Diseases; National Engineering Laboratory for Digital and Material Technology of Stomatology; Beijing Key Laboratory of Digital Stomatology, Beijing, Beijing, China. Conducted the experiments, analysed the results, reviewed the manuscript.; f Periodontist, Department of Periodontology, Peking University School and Hospital of Stomatology and National Clinical Research Center for Oral Diseases; National Engineering Laboratory for Digital and Material Technology of Stomatology; Beijing Key Laboratory of Digital Stomatology, Beijing, Beijing, China. Clinical data collection, reviewed the manuscript.; g Periodontist, Department of Periodontology, Peking University School and Hospital of Stomatology and National Clinical Research Center for Oral Diseases; National Engineering Laboratory for Digital and Material Technology of Stomatology; Beijing Key Laboratory of Digital Stomatology, Beijing, Beijing, China. Revised and reviewed the manuscript.; h Dentist, Department of Stomatology, Langfang Maternal and Child Health Center, Langfang, Hebei, China. Clinical data collection, reviewed the manuscript.

**Keywords:** dental care behaviours, periodontal health, periodontal pathogens, periodontal status, pregnancy

## Abstract

**Purpose:**

This study examines the difference in the oral microbiome during pregnancy and the postpartum period of a Chinese population, with the focus on *P. gingivalis, P. intermedia* and *P. nigrescens* and their shift during pregnancy, in order to understand the host-microbe relationship in maintaining homeostasis during pregnancy.

**Materials and Methods:**

This cross-sectional study was carried out in a total of 117 women who underwent prenatal or regular examinations at four public hospitals, including 84 pregnant and 33 postpartum women. Women in the postpartum group were examined within 0.5-1 year after delivery, while the pregnant group was divided into early pregnancy (0-13 weeks), middle pregnancy (14–27 weeks), and late pregnancy (28–39 weeks) according to gestational age. Sociodemographic parameters were self-reported by recruited women. The study required evaluations of probing depth (PD), bleeding index (BI), clinical attachment loss (CAL), and plaque index (PlI). Unstimulated whole saliva samples were collected for the detection of *P. gingivalis, P. intermedia,* and* P. nigrescens*. Bacterial populations were evaluated using 16S rRNA-based polymerase chain reaction.

**Results:**

*P. nigrescens* exhibited higher prevalence in the pregnant group compared to the postpartum group (45.67% vs 12.10%, p < 0.01). *P. nigrescens* was more frequently detected in late than in early pregnancy (57.7% vs 48.3%, p < 0.05) and middle pregnancy (57.7% vs 31.0%, p < 0.01). Initially high prevalence of *P. gingivalis* in early pregnancy wanes in middle and late pregnancy (69.0%, 44.8%, 38.5%). However, the prevalence of* P. gingivalis* in the postpartum group (81.8%) exceeds all of the pregnant groups (p < 0.01). The change in the prevalence of *P. intermedia* among different groups was not statistically significant. The percentages of bleeding on probing (BOP%) sites and PD ≥4 mm sites in the postpartum group were statistically significantly higher when compared with each of the pregnant groups (p < 0.01). During pregnancy, women experienced elevated PlI, BI, PD, and BOP% (p < 0.05). The proportions of subjects in the pregnant group who agreed with the statements ‘Gingival bleeding is normal’, ‘Can’t brush teeth within 1st month postpartum’, ‘It’s unnecessary to see a dentist if not uncomfortable’ were 39.3%, 28.6%, and 35.7%, respectively.

**Conclusions:**

*P. nigrescens* is more related than *P. gingivalis* to pregnancy status. The periodontal status of Chinese women progressively deteriorates during pregnancy and persists into the postpartum period, which may result from lack of dental care knowledge.

Periodontal diseases affect the gingiva and alveolar bone through pathological inflammation induced by chronic bacterial infection, or environmental, behavioural, and/or genetic factors.^38^ The oral health status of pregnant women is unsatisfactory in certain countries.^8^ Hormonal changes associated with pregnancy function as an environmental factor and aggravate pre-existing chronic gingival inflammation, inducing symptoms such as gingival bleeding and swelling.^23,26^ Elevated estrogen production throughout pregnancy indirectly affects periodontal tissue by potentially upregulating the growth of specific Gram-negative anaerobes. Experiments have confirmed that certain bacteria can utilise estrogen as an alternative to vitamin K in vitro to increase growth.^18^

Previously, *Prevotella intermedia* sensu lato (formerly *Bacteroides intermedius*) was widely considered a hormone-specific pathogen in gingivitis afflicting pregnant women.^19,25^ However, a notable revision of the classification of *Bacteroides* spp. due to advancements in molecular biology resulted in the formation of two new genera, *Porphyromonas* and *Prevotella*.^18,30^ Formerly classified as *Bacteroides intermedius, Prevotella* spp., *P. intermedia* and *P. nigrescens* are now identified as separate species despite a close phylogenetic relationship and indistinguishable phenotypes.^29^ Although many studies reported *P. intermedia* as the most common bacterium associated with the shift in pathogenic microbiota in pregnant women, these studies did not consider *P. nigrescens*.^7,17,19^ In 2009, Gürsoy et al^15^ reported *P. nigrescens* as the predominant bacterial species in pregnant women. Furthermore, only a few publications have suggested that *P. nigrescens* is related to pregnancy and provide evidence for a significant reduction postpartum.^22^

*P. gingivalis* is a well-known periodontal pathogenic bacterium related to inflammation and destruction of periodontal tissue. Moreover, elevation of progesterone and estrogen serum levels has been linked to *P. gingivalis*.^18^ The presence of *P. gingivalis* in gingival sulci is considerably elevated during the 1st and 2nd trimesters in comparison to non-pregnant women.^12^

In fact, the ecosystem, microbiotic composition, and structural changes of the oral cavity throughout gestation remain unknown. Current evidence is insufficient not only for determining the relationship between pregnancy and preferential bacterial colonisation, but also whether microbiota composition is consistent between different ethnic groups. Pregnant Chinese women fail to maintain daily dental care due to traditional values.^21^ Thus, whether the predominant pathogens in pregnant vs non-pregnant Chinese women differ still needs to be proven. By identifying the predominant bacteria affecting this population, this study aimed to more clearly elucidate microbiome changes throughout pregnancy by examining the difference in the oral microbiome during pregnancy and postpartum period of the Chinese population. The main focus was on *P. gingivalis,* *P. intermedia* and *P. nigrescens* and their shift during pregnancy, in order to understand the host-microbe relationship in maintaining homeostasis during pregnancy.

## Materials and Methods

This cross-sectional study was approved by the Peking University Biomedical Ethics Committee (IRB00001052-07104) and according to the principles outlined in the Declaration of Helsinki on experimentation involving human subjects. All recruited subjects provided written, informed consent of their participation.

### Study Subjects

Pregnant and postpartum women were recruited from gynecology clinics of four public hospitals from various geographical areas in Beijing, China, including two urban hospitals (the Third Hospital of Peking University, and Haidian Maternal and Child Health Hospital) and two suburban hospitals (Miyun Hospital and Miyun Maternal and Child Health Hospital) when they attended prenatal or postpartum examinations during 2007 and 2008. Their medical records were accessed by the investigator. Inclusion criteria were: 1. age between 20 to 40 years; 2. more than 24 natural teeth in the mouth excluding third molars; 3. systemically healthy (i.e. no diabetes, endocrine disorders, or hypertension); 4. primiparity. Exclusion criteria were: 1. acute endodontic or periodontal disease; 2. received periodontal treatments within the past 6 months; 3. took antibiotics or immunosuppressants in the previous 3 months; 4. smoking or alcohol habit; 5. multigravida; 6. gestational complications (i.e. pre-eclampsia). Additionally, women of the postpartum group were included if they were in 0.5-1 years postpartum and excluded if they were taking contraceptive drugs or planning to become pregnant again within the following six months. The gestation of pregnant women was divided into 1st trimester (0-13 weeks), 2nd trimester (14–27 weeks), and 3rd trimester (28-39 weeks). The weight of the newborns was also registered, and to be included they had to be delivered on term with standard weight.

Based on the data from our previous study,^16^ a difference of 0.36 mm probing depth was considered as a reference with statistical significance. Power was calculated with the Sample Power 2.0 program (IBM SPSS; Armonk, NY, USA), indicating that at least 26 subjects within each group would have 90% power to detect a 0.36-mm difference in probing depth during pregnancy with α set at 0.05. Therefore, taking into account potential drop-outs, sample size was established as 32 pregnant women for each group, 128 in total.

### Sociodemographic Features

Initially, every participant was asked to complete a self-reported questionnaire to determine their socioeconomic status and dental care awareness, including age, height, weight, family income, employment status, educational level, frequency of toothbrushing, dental care experience and frequency, and symptoms of their periodontal condition, etc. Lifestyle behaviours such as alcohol and tobacco preferences, medical history such as hypertension and diabetes, and a list of medications were also obtained. There was also a brief quiz to test their basic knowledge of periodontal health.

### Periodontal Clinical Examination

Full-mouth (excluding the third molars) periodontal examinations were performed by 3 examiners (ZH, LH, JK), using a Williams periodontal probe (Hu-Friedy; Chicago, IL, USA). To test the validity of periodontal clinical measurements, all examiners were previously calibrated with a senior examiner (YS) in a pilot study of 10 subjects, until intra- and inter-examiner reliability of no less than 88% (Cohen’s Kappa Test) was achieved. Clinical probing depth (PD) was obtained from six sites per tooth (mesial, distal, and middle sites of the buccal and lingual sides). The maximum bleeding index (BI) values were measured at the buccal and lingual surfaces 30 s after probing into the bottom of sulcus or pocket.^24^ A binary index scored as 0 or 1 based on the absence or the presence of bleeding, respectively, was used to evaluate bleeding on probing (BOP) on two surfaces per tooth. The highest plaque index (PlI)^31^ of the buccal and lingual surfaces was recorded. To calculate visible plaque index (VPI), visible bacterial plaque was measured on two surfaces per tooth using a binary index as previously described and the surfaces with plaque were recorded as a percentage of total surfaces evaluated.^1^ Clinical attachment loss (CAL) was measured by the sum of PD and gingival recession on Ramfjord teeth^28^ with six sites per tooth (mesial, distal, and middle sites of the buccal and lingual sides).

### Sample Collection and Processing

The protocol used was based on Feng et al.^9^ Prior to probing, unstimulated whole saliva was obtained. 0.5 ml of saliva (sample processing was completed within 4 h) was transferred to a 1.5-ml Eppendorf tube for centrifugation (5 min at 13,000 rpm, rotor radius = 5.5 cm). After carefully discarding the supernatant, the pellet was thoroughly dispersed in 0.5 ml of TE buffer. Then, the centrifugation described above was repeated (5 min at 13,000 rpm, rotor radius = 5.5 cm). After washing with 0.5 ml of TE buffer four times, the precipitate was frozen at -20°C for DNA extraction.

### DNA Extraction and PCR Measurements

A commercial bacterial DNA extraction kit provided by Shanghai Huashun Bioengineering (Shanghai, China) was used to extract the DNA. In accordance with Ashimoto et al^2^ and Baumgartner et al,^4^ 16S rDNA primers for *P. gingivalis, P. intermedia* and *P. nigrescens* were designed (Table 1). The final PCR reaction system of 25 μl containing 2 μl of sample, 10 mmol/l Tris-HCL, 50 mmol/l KCL, 0.2 mmol/l dNTP, 0.4 μmol/l primer, 1U Taq DNA polymerase, 1.5 mmol/l MgCl_2_ for *P. gingivalis* samples, and 1.0 mmol/l MgCl_2_ for *P. intermedia* and *P. nigrescens* samples.

**Table 1:** Species-specific PCR primers for periodontal pathogen detection

Strain	Oligonucleotides (5’→3’)	Amplicon size (bp)
*P. gingivalis*	AGG CAG CTT GCC ATA CTG CG ACT GTT AGC AAC TAC CGA TGT	404
*P. intermedia*	TTT GTT GGG GAG TAA AGC GGG TCA ACA TCT CTG TAT CCT GCG T	575
*P. nigrescens*	ATG AAA CAA AGG TTT TCC GGT AAG CCC ACG TCT CTG TGG GCT GCG A	804

A GeneAmp PCR system 2700 instrument (Thermo Fisher Scientific; Waltham, MA, USA) was used to perform PCR reactions. The reaction cycles for *P. gingivalis* samples comprised 2 cycles of pre-denaturation at 95°C, 36 cycles of denaturation at 95°C for 30 s, annealing at 60°C for 1 min, extension at 72°C for 1 f and a final extension at 72°C for 2 min. In contrast, the PCR reaction cycles for *P. intermedia* and *P. nigrescens* samples included pre-denaturation at 95°C for 2 min, then 36 cycles of denaturation at 94°C for 30 s, annealing at 55°C for 1 min, extension at 72°C for 2 min, and finally an extension at 72°C for 10 min after the 36th cycle. Using a 1.5% agarose gel, the PCR products were visualised using gel electrophoresis.

The gel was stained with 50 μl/l nucleic acid stain (GoldView, SBS Genetech; Beijing, China) and photographed under 300-nm ultraviolet light. A 100-bp DNA ladder (DL 2,000 100-bp ladder, TaKaRa Biotechnology; Dalian, China) served as the molecular weight marker.

Positive and negative controls were used as quality controls for each PCR reaction. DNA extract from the respective microbe was used as the positive control of *P. gingivalis* (ATCC 33277), *P. intermedia* (ATCC 25611), and *P. nigrescens* (ATCC 33563), with the standard strain supplied by Tokyo Medical and Dental University and Capital Medical University Institute of Stomatology. The negative control for each was sterile, deionized water. After randomised selection, 50% of samples were re-tested, replicating 97.3% of the results. For the 2.7% of results that were conflicting, a third PCR reaction was performed.

### Statistical Analysis

All statistical analyses were performed using the SPSS 19.0 (Chicago, IL, USA) software package. Data were reported as mean ± SD or as a percentage. The normally distributed data were analysed with one-way ANOVA and the t-test. Quantitative data were compared using the chi-squared test. Pathogen prevalence differences between different groups were tested by the chi-squared test. One-way ANOVA was used to determine statistically significant differences of periodontal clinical status among the three groups during pregnancy, while the unpaired Student’s t-test was used to compare the differences between pregnancy and the postpartum period.

## Results

### Study Subjects

A total of 128 women were requested to partake in our study, 11 of them withdrew during the study due to personal reasons such as no time or no interest. Finally, 117 women complied with all of the terms required. Of the 117 women recruited, 84 women were pregnant, and 33 women were postpartum. The average age in the pregnant group was 29.11 ± 3.85 years, ranging from 21 to 38 years old. The average age of postpartum group was 29.76 ± 3.42 years, ranging from 23 to 38 years old (Table 2). All infants were delivered on term (mean 38.8 ± 1.37 weeks; range 37–41 weeks) with standard weight (mean 3127 ± 396 g; range, 2578–4186 g). All subjects in this study were generally healthy.

**Table 2:** Demographic characteristics of study subjects

		Pregnant (n=84)	Postpartum (n=33)	p-value
Age (years)	Mean ± SD	29.11 ± 3.85	29.76 ± 3.42	NS^a^
	Range	21 – 38	23 – 38	
Height (cm)	Mean ± SD	160.42±4.65	161.12±4.12	NS^a^
	Range	152 – 174	150 – 168	
Weight (kg)	Mean ± SD	57.27±11.15	57.03±9.54	NS^a^
	Range	45 – 75	42 – 90	
Family income per month (in RMB)	<3000	60.71%	45.45%	NS^b^
	>3000	39.29%	54.55%	
Employment	Employee	57.14%	81.82%	0.018^b^
	Housewife	42.86%	18.18%	
Level of education (years)	0-12	57.14%	36.36%	NS^b^
	>12	42.86%	63.64%	
Daily toothbrushing frequency during pregnancy	Twice or more	75.47%	81.82%	NS^b^
	Once	22.10%	18.18%	
	Never	2.43%	0.00%	
Toothbrushing duration during pregnancy (min)	<2	72.62%	63.64%	NS^b^
	2 or more	27.38%	36.36%	
Oral treatment before pregnancy	yes	11.90%	27.27%	NS^b^
	never	88.10%	72.73%	
Self-report of oral status during pregnancy	Gingival bleeding	45.45%	40.48%	NS^b^
	Tooth mobility	3.57%	0.00%	NS^b^
	Gingival swelling	18.18%	9.52%	NS^b^
Misconception of oral health	Gingival bleeding is normal	39.29%	24.24%	NS^b^
	Can‘t brush teeth within 1st month postpartum	28.57%	24.24%	NS^b^
	It‘s unnecessary to see dentist if not uncomfortable	35.71%	21.21%	NS^b^

^a^statistical significance determined using unpaired Student’s t-test; ^b^statistical significance determined using the chi-squared test; NS: not significant.

### Sociodemographic Characteristics

The sociodemographic information of pregnant participants and postpartum controls are shown in Table 2. Of the pregnant participants, 2.4% reported never brushing their teeth, 22.1% reported brushing only once per day, 72.6% reported brushing for less than 2 min, and 88.1% reported having never been to a clinic for periodontal health care. Regarding the questions about oral symptoms, 45.5% of the pregnant women experienced ‘gingival bleeding’, 3.8% experienced ‘tooth mobility’, and 18.2% experienced ‘gingival swelling’. The questionnaire revealed misconceptions of oral health: 39.3%, 28.6%, and 35.7% of subjects in the pregnant group agreed with the following statements, respectively, ‘Gingival bleeding is normal’, ‘Can’t brush teeth within the first month postpartum’, ‘It’s unnecessary to see dentist if not uncomfortable’. Subjects did not receive periodontal health care either during or after pregnancy. There were no significant differences in the sociodemographic variation between pregnant and postpartum groups (Table 2).

### Periodontal Status

#### Changes during pregnancy

As pregnancy progressed, the full-mouth PlI and percentage of sites with VPI increased. The percentage of sites with VPI was recorded as 35.6%, 51.9%, 58.2% in the 1st, 2nd and 3rd trimester, respectively (p < 0.001). The full-mouth mean BI (p = 0.013) and the percentage of sites with BOP (p = 0.011) also increased significantly. Similarly, there was an increase in mean PD from 1.74 ± 0.36 mm in the 1st trimester to 2.19 ± 0.18 mm in the 3rd trimester (p < 0.001). However, the percentage of PD over 4 mm in the 2nd trimester is significantly higher than the other groups (p = 0.016). There was no significant difference in CAL between the three groups (Table 3).

**Table 3:** Comparison of periodontal clinical status in different groups

		1st trimester (n=29)	2nd trimester (n=29)	3rd trimester (n=26)	p-value^a^	Pregnant (n=84)	Postpartum (n=33)	p-value^b^
PlI	Mean±SD	1.39 ± 0.25	1.66 ± 0.48	1.77 ± 0.49	0.004	1.60 ± 0.44	1.72 ± 0.47	NS
Site with VPI (%)	Mean±SD	35.55 ± 18.22	51.89 ± 23.54	58.15 ± 18.70	0.000	48.18 ± 22.27	54.96 ± 22.03	NS
	Range	28.62 ~ 42.48	42.93 ~ 60.84	50.60 ~ 65.70		3.57 ~ 100.00	47.14 ~ 62.77	
BI	Mean±SD	1.07 ± 0.67	1.32 ± 0.77	1.64 ± 0.62	0.013	1.33 ± 0.72	2.34 ± 0.92	0.000
Site with BOP (%)	Mean±SD	33.44 ± 23.28	40.62 ± 27.45	54.98 ± 27.06	0.011	42.61 ± 27.14	65.86 ± 29.34	0.000
	Range	0.00 ~ 92.86	0.00 ~ 92.86	7.14 ~ 100.00		0.00 ~ 100.00	3.57 ~ 100.00	
PD (mm)	Mean±SD	1.74 ± 0.36	2.07 ± 0.58	2.19 ± 0.18	0.000	1.99 ± 0.45	2.35 ± 0.42	0.000
Site with PD≥4 mm (%)	Mean±SD	1.41 ± 3.64	5.69 ± 7.64	2.97 ± 3.99	0.016	3.32 ± 5.60	6.72 ± 9.72	0.021
	Range	0.00 ~ 15.28	0.00 ~ 29.76	0.00 ~ 16.67		0.00 ~ 30.00	0.00 ~ 42.86	
CAL (mm)	Mean±SD	0.39 ± 0.26	0.39 ± 0.29	0.45 ± 0.41	NS	0.41 ± 0.32	0.22 ± 0.38	0.006

^a^statistical significance determined using one-way ANOVA; ^b^statistical significance determined using unpaired Student’s t-test.

#### Comparison between pregnant and postpartum women

Compared with pregnant women, in postpartum women, mean BI (p < 0.001), percentage of sites with BOP (p < 0.001), mean PD (p < 0.001), and percentage of sites with PD > 4 mm (p = 0.021) were statistically significantly higher. CAL in the postpartum group was statistically significantly lower than in the pregnant group (p = 0.006). There was no statistically significant difference in PlI and percentage of sites with VPI between two groups (Table 3).

### Microbiological Results

#### Frequency of detection of pathogens

Table 4 shows the prevalence of each periodontal pathogen from the pregnant women in three groups and postpartum women. The detected pathogens during pregnancy were *P. gingivalis* (range 38.5%–69%), *P. nigrescens* (range 31%–57.7%) and *P. intermedia* (range 13.8–46.2%). The prevalence of *P. gingivalis* gradually decreased as gestation progressed (69%, 44.8%, 38.5% respectively) but was statistically significantly higher in the postpartum group (81.8%) than in any stage of pregnancy (p = 0.002). The prevalence of *P. intermedia* was neither statistically significantly different between the three pregnant groups nor between pregnant and postpartum groups. The prevalence of *P. nigrescens* in the postpartum group (12.1%) was significantly lower than in all three pregnant groups (48.3%, 31%, 57.7%) (p = 0.001). Between pregnant groups, the prevalence of *P. nigrescens* in the 3rd trimester was significantly higher than in the other two trimesters (p = 0.001).

**Table 4:** Frequency of detection of periodontal pathogens (percentage/absolute number related to group)

	1st trimester (n=29)	2nd trimester (n=29)	3rd trimester (n=26)	Postpartum (n=33)	p-value^a^
*P. gingivalis*	69.00% (20)	44.80% (13)	38.50% (10)	81.80% (27)	0.002
*P. intermedia*	27.60% (8)	13.80% (4)	46.20% (12)	24.20% (8)	NS
*P. nigrescens*	48.30% (14)	31.00% (9)	57.70% (15)	12.10% (4)	0.001

^a^Statistical significance determined using chi-squared test.

### Periodontal Clinical Parameters in Patients Harbouring Periodontal Pathogens

To evaluate the microbiological differences in a certain clinical condition, patients were placed into subgroups based on the presence or absence of a specific pathogen. As illustrated in Table 5, *P. gingivalis*-positive participants exhibited increases in all of the periodontal clinical indices of PlI, the percentage of sites with BOP, PD >4 mm, and CAL, as opposed to *P. gingivalis*-negative participants in all groups. However, there was no statistically significant change in periodontal clinical indices between positive and negative subjects affected by *P. intermedia* or *P. nigrescens*.

**Table 5:** Clinical parameters according to the presence [positive patients (+)]/absence [negative patients (−)] of *P. gingivalis,* *P. intermedia* and *P. nigrenscens* (mean±SD)

			Pregnant (n=84)	Postpartum (n=33)	
Site with VPI (%)	*P. gingivalis*	+	48.58 ± 22.29	58.50 ± 22.70	*
		-	47.77 ± 22.53	38.99 ± 7.27	
	*P. intermedia*	+	49.50 ± 21.96	70.78 ± 20.39	*
		-	47.66 ± 22.56	49.89 ± 20.39	
	*P. nigrenscens*	+	45.27 ± 18.75	70.62 ± 11.41	*
		-	50.60 ± 24.75	52.80 ± 22.38	
Site with BOP (%)	*P. gingivalis*	+	44.42 ± 25.51	70.70 ± 26.14	*
		-	40.71 ± 28.95	44.00 ± 34.91	
	*P. intermedia*	+	41.21 ± 29.18	65.50 ± 27.75	
		-	43.17 ± 26.52	65.96 ± 30.26	
	*P. nigrenscens*	+	44.89 ± 25.52	62.25 ± 32.16	
		-	40.72 ± 28.55	66.34 ± 29.40	
Site with PD ≥ 4 mm (%)	*P. gingivalis*	+	3.77 ± 6.49	8.11 ± 10.29	*
		-	2.85 ± 4.50	1.00 ± 1.10	
	*P. intermedia*	+	3.04 ± 5.74	11.88 ± 10.45	*
		-	3.43 ± 5.58	5.20 ± 9.06	
	*P. nigrenscens*	+	2.18 ± 3.21	7.75 ± 5.12	
		-	4.26 ± 6.87	6.69 ± 10.22	
Site with CAL (%)	*P. gingivalis*	+	31.52 ± 19.66	16.67 ± 20.99	*
		-	25.88 ± 17.07	1.85 ± 2.27	
	*P. intermedia*	+	29.51 ± 19.74	21.18 ± 22.17	
		-	28.47 ± 18.22	11.67 ± 18.89	
	*P. nigrenscens*	+	27.34 ± 19.43	19.44 ± 21.52	
		-	29.95 ± 17.92	13.22 ± 19.85	

^a^Statistical significance determined using unpaired Student’s t-test; *p<0.05 (comparison between positive and negative groups).

## Discussion

The present study provides periodonto-pathogenic microbial data and clinical results as a basis for understanding the aetiology of pregnancy-associated oral dysbiosis and its impact on periodontal status. We observed a higher prevalence of *P. nigrescens* in the saliva of the pregnant group than in the postpartum group, while the prevalence of *P. gingivalis* and local inflammation in the postpartum group statistically significantly exceeded each of the pregnant groups. These data indicate that *P. nigrescens* is more connected to pregnancy status than is *P. gingivalis*. Additionally, we found that periodontal status of Chinese women progressively deteriorates during pregnancy and persists into postpartum, which may corelated with a lack of dental care knowledge.

With increased hormone levels during pregnancy, the response of gingival tissue to plaque stimulation may increase and pre-existing inflammation may become exacerbated. In the weeks leading up to delivery, estrogen and progesterone levels increase dramatically, peaking in the 3rd trimester.^11^ In this study, as pregnancy progressed, PD and the percentage of sites with BOP increased and peaked during the 3rd trimester. Therefore, these changes may stem from pregnancy-related elevation of hormones and exacerbation of gingivitis. The periodontal status of different subjects in different pregnant groups are consistent with the characteristics of the population and the findings of previous studies,^14,37^ which showed a gradual increase in gingival inflammation from the 1st to 3rd trimester.

Intriguingly, although hormone levels decrease postpartum,^11^ the clinical results of BI and PD persisted and increased in postpartum women, indicating periodontal inflammation was more serious. These results deviate from earlier reports of cross-sectional studies.^34,36^ The sociodemographic characteristics of our study population presented above demonstrate that most subjects have poor dental care awareness and hold misconceptions about oral health. It can be deduced that they rarely seek periodontal care. Moreover, they may have suffered from gingivitis before pregnancy and experienced increased severity of gingivitis during pregnancy. If plaque factors were not eliminated, more subjects would have presented with periodontal disease. Since some women did not brush their teeth within the first month after delivery-postpartum periodontal inflammation worsened instead of subsided as reported.^34,36^ If such a condition progresses continuously without any treatment, the periodontal status may deteriorate and cause attachment loss. However, in the present study CAL was slightly lower in the postpartum group. This should be attributed to the limitation of the study: it is a cross-sectional study among four groups of subjects instead of a longitudinal study in a cohort population with a larger sample size. Periodontal inflammation worsened after delivery, indicating increased risk of attachment loss in the future. Therefore, we concluded that the postpartum women had worse periodontal status in the present study.

During pregnancy, the increased vascular permeability and oedema of gingival tissue lead to an increased sulcus depth, providing a more receptive microenvironment for the growth of anaerobic gram-negative bacteria. However, the effect of pregnancy status on changes of periodontal pathogens and the effect of the corresponding microbiological ecosystem on the periodontal condition of pregnant and postpartum women remain unclear. With improved taxonomic methods and molecular biology, contemporary research provides an updated and more refined perspective on oral microbiota changes during pregnancy.^3,20^ This work supports the role of elevated sex steroids throughout pregnancy as a modulator of the human oral microbiota.

As a red complex, *P. gingivalis* was connected to periodontitis and periodontal clinical parameters, especially in deep pockets and gingival bleeding sites.^32^ Here, the prevalence of *P. gingivalis* in the postpartum group was statistically significantly higher than in all pregnant groups. Furthermore, VPI, PD, and BOP in the postpartum group were significantly higher as well, consistent with the characteristics of *P. gingivalis*. Moreover, a noteworthy result was the considerable increase in VPI, BOP, and the percentage of sites with PD >4 mm in *P. gingivalis-*positive women in the postpartum group, which demonstrated *P. gingivalis*’s association with more severe periodontal inflammation. Consistent with another study,^12^ *P. gingivalis* levels in the present study were significantly higher during the 1st and 2nd trimester. However, in the 3rd trimester, the prevalence of *P. gingivalis* decreased while *P. nigrescens* became the most abundant bacterium.

*P. intermedia* and *P. nigrescens* are both orange complexes associated with periodontitis progression. Like the red complex, the orange complex is linked to periodontal pocket depth. Red and orange complexes are closely related, contributing immensely to the diagnosis of periodontal disease. The present study finds that the percentage of VPI and PD >4 mm increased statistically significantly in *P. intermedia*-positive women in the postpartum group, consistent with the characteristics of *P. intermedia*. However, the presence or absence of the pathogen yielded no substantial variations in clinical parameters between pregnant women (Table 5). Hormonal changes during pregnancy likely influence the expected correlation between the periodontal indices and the presence of the pathogen.

Due to the shortage of conventional phenotypic biochemical methods, previous studies on *P. intermedia* showed contradictory results. Our results corroborate some earlier findings,^22^ and further explain the findings concerning *P. intermedia* and *P. nigrescens* in the oral cavity of pregnant women. These findings suggest that *P. nigrescens* is the related pathogen in gingivitis during pregnancy, and not *P. intermedia* as previously believed.^17,19^ *P. nigrescens* is mediated by hormonal and metabolic changes during pregnancy, such as elevation of estrogen levels, suppressed immune responses, and a suitable environment for bacterial growth. Thus, the increase of *P. nigrescens* throughout gestation may be attributed to elevated progesterone since progesterone can substitute for vitamin K in *P. nigrescens*. The ensuing decrease of *P. nigrescens* in the postpartum period when progesterone levels declined supports this conclusion.^19^ Therefore, elevated hormone levels during pregnancy lead to increases in the amount of *P. nigrescens*. The detection rate of *P. nigrescens* during pregnancy was notably elevated when compared to the postpartum period (Table 4). A Finnish study published similar results.^15^ *P. nigrescens* may affect pregnant women suffering from gingivitis, irrespective of race or diet.

In our study, salivary bacteria were examined instead of supra/subgingival biofilm, since microbes in saliva originate from the sulci, tongue dorsum, and other regions in the oral cavity. Results commonly reflected oral infection among individual patients. It has been reported that the salivary microbiome can reflect the periodontal condition.^5^ A study concluded that in regard to detecting *P. gingivalis*, a sample of whole saliva seems to be superior to a pooled periodontal pocket sample.^35^ In our lab, we demonstrated a 100% duplication of *P. gingivalis* detection in saliva and pooled subgingival samples in severe periodontitis, while detection rates improved slightly using saliva as opposed to pooled subgingival samples in non-periodontitis subjects.^10^ In addition, all the pathogens in periodontal pockets can be enter the saliva via gingival crevicular fluid, and detection in saliva is positively related to the number of deep pockets.^10^ Conversely, the subgingival microbiome is largely dependent on periodontal depth and local inflammation of individual sites.^13^ The gingival crevicular fluid or supra/subgingival biofilm samples collected from a certain tooth might be more site-specific. Thus, whole saliva samples may have an accumulation of bacteria as opposed to a single periodontal pocket sample.^35^ Therefore, saliva may be a practical substitute for pooled supra/subgingival biofilms when detecting periopathogens in participants.^5^

Recruiting participants from the four selected recruitment centers in various geographical areas, 2 urban and 2 suburban hospitals, was intended to represent a broad range of demographic characteristics of the Chinese population. The self-reported sociodemographic questionnaire revealed socioeconomic status, dental care awareness, self-reported oral symptoms related to periodontal condition and lifestyle behaviour among Chinese women during pregnancy and 0.5-1 year postpartum. Although many studies suggest that pregnant women who develop gingivitis may experience a reduction or absence of symptoms postpartum,^23^ the questionnaire in present study showed most women’s self-reported periodontal condition worsened during pregnancy and 0.5-1 year postpartum. This may be due to traditional perceptions and poor oral hygiene awareness among Chinese women, which can be shown from our survey (Table 2). ‘Can’t brush teeth within first month postpartum’ is an old traditional misconception, which still negatively affects young mothers today. Additionally, poor oral hygiene awareness may be caused by insufficient hygiene instructions. A previous study reported 11.2% of women sought treatment from their dental practitioner after recognising changes in their oral condition,^33^ and only 1 out of 4 women is informed by their gynecologist about the importance of regular dental check-ups during pregnancy.^27^ Such a low rate of oral-hygiene information transfer to patients is probably related to gynecologists being less informed about periodontal diseases, reducing the likelihood of a timely diagnosis.^27^ Disappointingly, prenatal care practitioners were unaware of periodontal diseases during pregnancy, which indicates an absence of appropriate continuing education in the field.^6^

Although the reason remains unclear, it can be concluded from the present study that *P. nigrescens* is more related to pregnancy status than *P. gingivalis* and *P. intermedia*. Throughout gestation, the periodontal status of Chinese women progressively deteriorated. Moreover, periodontal status remained unchanged or slightly worsened in women within one year after delivery. Collectively, without correct knowledge of dental care and treatment, gingivitis will not subside postpartum as hormone levels return to normal, verifying the value of dental care throughout pregnancy and the postpartum period. Therefore, a sizable shift in the perception of gingivitis in pregnancy among doctors and patients is required to reverse these alarming data.

In addition, although the sociodemographic characteristics of the pregnant and postpartum women in this study are very similar, it is not the longitudinal observation of a cohort population but a cross-sectional study among comparable subjects. This is a shortcoming that may limit our conclusion. Other limitations were the relatively small sample size, so that the difference between individuals may have a relevant impact the results.

Are the changes in the composition of the subgingival biofilm determined by the microbiological shift in the bacterial ecosystem or rather by the genetically or epigenetically determined tissue structures? Do the altered, innate, and adaptive immune responses disturb the composition of the normally commensal flora? A longitudinal study in a cohort population is needed to comprehensively answer these questions.

## Conclusions

Our study demonstrates *P. nigrescens* is more related to pregnancy status than are the other pathogens. The periodontal status of Chinese women progressively deteriorates during pregnancy and persists into the postpartum period under the conditions established by insufficient knowledge of oral health care. The data suggest that a physiological change during pregnancy has a significant impact on certain bacteria in the oral cavity and may affect periodontal status. For pregnant women in China, periodontal health programs are indispensable in preventing periodontal disease.
